# Balanced bilingualism and early age of second language acquisition as the underlying mechanisms of a bilingual executive control advantage: why variations in bilingual experiences matter

**DOI:** 10.3389/fpsyg.2015.00164

**Published:** 2015-02-26

**Authors:** W. Quin Yow, Xiaoqian Li

**Affiliations:** Humanities, Arts and Social Sciences, Singapore University of Technology and DesignSingapore

**Keywords:** executive control, bilingualism, age of acquisition, language usage, language proficiency

## Abstract

Recent studies revealed inconsistent evidences of a bilingual advantage in executive processing. One potential source of explanation is the multifaceted experience of the bilinguals in these studies. This study seeks to test whether bilinguals who engage in language selection more frequently would perform better in executive control tasks than those bilinguals who engage in language selection less frequently. We examined the influence of the degree of bilingualism (i.e., language proficiency, frequency of use of two languages, and age of second language acquisition) on executive functioning in bilingual young adults using a comprehensive battery of executive control tasks. Seventy-two 18- to 25-years-old English–Mandarin bilinguals performed four computerized executive function (EF) tasks (Stroop, Eriksen flanker, number–letter switching, and *n*-back task) that measure the EF components: inhibition, mental-set shifting, and information updating and monitoring. Results from multiple regression analyses, structural equation modeling, and bootstrapping supported the positive association between age of second language acquisition and the interference cost in the Stroop task. Most importantly, we found a significant effect of balanced bilingualism (balanced usage of and balanced proficiency in two languages) on the Stroop and number–letter task (mixing cost only), indicating that a more balanced use and a more balanced level of proficiency in two languages resulted in better executive control skills in the adult bilinguals. We did not find any significant effect of bilingualism on flanker or *n*-back task. These findings provided important insights to the underlying mechanisms of the bilingual cognitive advantage hypothesis, demonstrating that regular experience with extensive practice in controlling attention to their two language systems results in better performance in related EFs such as inhibiting prepotent responses and global set-shifting.

## INTRODUCTION

Executive control refers to the set of skills required for cognitive processes such as inhibition, switching attention, and working memory. Research suggests that bilingualism confers advantages in executive control across the life-span (e.g., Bialystok and Martin, 2004; Bialystok et al., 2008; Carlson and Meltzoff, 2008; Morales et al., 2013, see Adesope et al., 2010; Hilchey and Klein, 2011 for a recent review of children and adults studies respectively). Researchers argue that bilinguals show parallel activation of both of their languages as well as some interaction between these languages (e.g., Marian et al., 2003; Thierry and Wu, 2007). Consequently, bilinguals have to constantly monitor and control attention to the correct desired language system, instead of the competing other language, in order to stay relevant in the communication process. The processes required in the linguistic and non-linguistic processing of bilinguals are argued to be the same set of cognitive processes recruited for general executive functioning, hence, resulting in better executive control skills in bilinguals compared to monolinguals (Green, 1986, 1998; Rodriguez-Fornells et al., 2002; Colzato et al., 2008; Bialystok and Viswanathan, 2009; Bialystok et al., 2012). Neuroimaging studies also provided converging evidence that the cortical regions underlying general executive functioning, such as dorsolateral prefrontal cortex, left inferior frontal gyrus, left middle temporal gyrus, and anterior cingulated cortex, are involved in bilingual language-switching and dual language processing (e.g., Hernández et al., 2000; Abutalebi and Green, 2008; Toro et al., 2008; Hedden and Gabrieli, 2010; Rubio-Fernández and Glucksberg, 2012).

A careful tracing of prior literature reveals a picture far from simple, however. Some research discovered that bilingualism aids executive control with a smaller cognitive cost (Costa et al., 2008; Hernández et al., 2012), while others found a bilingual advantage in executive control skills as an overall speed advantage of bilinguals in response times (Bialystok et al., 2004; Bialystok and Viswanathan, 2009). Yet other research supports a bilingual advantage in some cognitive systems or tasks but not in others (Costa et al., 2009; Hernández et al., 2010, 2013). For example, Costa et al. (2009) argued that a bilingual advantage in ignoring distracting information was present only when the flanker task required high-monitoring resources but not when it required low-monitoring resources. Hernández et al. (2010) found a bilingual advantage in the executive control network of attention (Stroop task) but not in the orienting network of attention (visual cueing task). Furthermore, a separate set of studies failed to show a bilingual advantage in executive functioning at all (Paap and Greenberg, 2013; Paap and Sawi, 2014). Recent reviews of the literature concluded that there is inconsistent evidence for a bilingual advantage in executive processing. This conflicting state of the literature is suggested to have stemmed from the lack of clarity in how executive functions (EFs) are defined and measured, the lack of control over factors that may modulate EF, as well as the lack of clarity in how bilingualism is defined and measured (e.g., Hilchey et al., in press; see Valian, 2015, for a more recent review).

Regardless of whether significant results of a bilingual advantage were found or not, or under what conditions using what tasks, past research examining the effects of bilingualism on executive control has focused on comparing groups of monolingual and bilingual individuals, treating the two groups as equally distinct from each other while considering the group members as a homogenous whole. Oftentimes, individuals from the two language groups are drawn from populations that differ in demographics, such as different nationalities and different language families, making the comparisons difficult to interpret. In addition, past studies tend to categorize bilinguals who differ on multiple dimensions into distinct bins, e.g., early vs. late, simultaneous vs. sequential, more proficient vs. less proficient, L1-dominant vs. L2-dominant, balanced vs. unbalanced, active vs. passive, etc. However, bilingualism is a dynamic experience that is composed of multiple dimensions and should not be considered as a discrete variable (Luk and Bialystok, 2013; Kaushanskaya and Prior, 2015; Luk, 2015). Different types of bilingual experience may affect the development of executive control in different ways and to different extents.

Thus, generalizing arguments around a cognitive advantage in bilinguals over monolinguals without considering the heterogenetic nature of bilingualism can lead to potentially flawed conclusions. If the underlying mechanism of a proposed bilingual cognitive advantage found by some studies is the frequent engagement in control and attention to the appropriate language system, then bilinguals who engage in language selection more frequently should exhibit an advantage in executive control tasks over those bilinguals who engage in language selection less frequently. Testing how variations in language practices and exposure among the bilinguals would affect their executive control skills would be critical to establishing the validity of the bilingual cognitive advantage hypothesis.

Few studies have investigated the effects of bilingualism on executive control within the bilingual population exclusively. Soveri et al. (2011), for example, investigated the effects of language background factors such as language-switching, use of both languages, and age of second language (L2) acquisition on EF components with 38 Finnish–Swedish bilinguals aged between 30 and 75-years-old. The bilinguals were tested on four tasks consisting of Simon, flanker, spatial *n*-back, and number–letter task switching. Multiple regression analyses found an effect of age of acquisition (AoA) on prepotent response inhibition: a younger age of L2 acquisition resulted in a smaller Simon effect. Participants who acquired their second language at a younger age and used both languages equally also performed better in task switching with lower mixing cost. However, the study involved bilinguals of a wide age range and it is not known whether the effects of L2 AoA and language usage on executive functioning were derived mainly from participants of a specific age group. Executive control abilities develop from infancy, peak at young adulthood, and decline at old age. Individual differences in executive control skills can be more easily detected in children and the elderly compared to young adults (Craik and Bialystok, 2006; Davidson et al., 2006).

Indeed, past studies found an executive control advantage in the bilingual elderly compared to the monolingual elderly, but results with the young adults were mixed (e.g., Costa et al., 2009; Paap and Greenberg, 2013). For example, Bialystok et al. (2005b) reported a bilingual advantage in reaction time on the Simon task in 5-years-old and older adults (60–80-years-old) but not the younger adults (30–59-years-old). Bialystok et al. (2008) found a bilingual advantage in the Stroop task in younger and older adults but when the same participants performed the Simon arrow task, the bilingual advantage was found only in the older adults. Therefore, it is possible that participants from a specific age group drove the significant effects of AoA and language usage on the performance of EF tasks in Soveri et al.’s (2011) study.

In another study, Luk et al. (2011) studied the relationship between onset age of bilingualism and cognitive control, comparing early bilinguals (those who started active bilingualism before 10-years-old), late bilinguals (an onset age of active bilingualism after 10-years-old), and monolinguals in an adapted flanker task. They found that early bilinguals produced the smallest response time cost for incongruent trials (flanker effect), with monolinguals and late bilinguals performing at similar levels. In addition, they found that the onset age of active bilingualism was negatively correlated with English proficiency and positively correlated with the flanker effect. They concluded “more experience in being actively bilingual is associated with greater advantages in cognitive control and higher language proficiency” (p. 588). However, the study did not distinguish between duration of being bilingual, L2 AoA and language proficiency; as such it remains possible that the early bilinguals turned out to be more proficient in their L2 earlier than the late bilinguals (Luk et al., 2011; Iluz-Cohen and Armon-Lotem, 2013). In addition, the authors categorized bilingualism into discrete groups based on an onset age of 10, but it is worthy to note that this cut-off age of 10 remains somewhat arbitrary. Bilingualism should still be best regarded as a characteristic, ability, or behavior that falls along a continuum.

In a most recent study by Pelham and Abrams (2014), researchers compared three groups of participants in an attentional network task (ANT): English monolinguals, early Spanish–English bilinguals who became fluent in L2 before 7 years of age, and late Spanish–English bilinguals who became fluent in L2 no earlier than 13-years-old. The results showed equivalent executive control benefits in the early and late bilinguals compared with the monolinguals. The researchers thus claimed that the benefits of bilingualism for executive control, specifically the inhibition of interference from distractors, were a result of the habitual use of two languages with limited influence from the length of time being proficient in both languages. Note that the bilingual individuals in their study were grouped into distinct groups based on a criterion that was different from that used in Luk et al.’s (2011) study. The early and late bilinguals in Pelham and Abrams’s study were equivalent in the percentage of time spent speaking their dominant or non-dominant languages. Both groups of bilinguals could also be considered as proficient bilinguals (i.e., using a 10-point scale, even the late bilinguals had an average score of 8.4 for understanding their non-dominant language). As a result, the equivalent executive control benefits for the early and late bilinguals could, too, be due to their comparable proficiency in their two languages. This study suggests that variables of bilingualism, such as language usage and language proficiency, are important factors when investigating the effects of bilingualism on executive functioning.

In sum, the bilingual cognitive advantage hypothesis suggests that bilinguals derive cognitive benefits from maintaining control and attention to the appropriate language system. However, the extent of these cognitive benefits should be dependent on factors that influence the exposure and opportunity to practice monitoring and controlling attention to the two language systems. For example, the more a bilingual uses the two languages, the more proficient he/she will get in both languages. As the bilingual gains proficiency in each of the languages and uses both languages regularly, he or she will have to exert more control and attention to prevent intrusion from the inappropriate language system. Similarly, early acquisition of two languages provides early exposure to and more time for a bilingual to practice using and controlling the two languages. Therefore, if frequent practice of controlling and attending to the appropriate language system confers general advantage in executive control tasks, then *balanced bilingualism* (defined here as both a balanced use and a balanced level of proficiency in two languages), in addition to early dual language acquisition, would critically affect the development of executive control skills in bilinguals. Thus, the first goal of our study is to treat bilingualism as a continuous variable and test the bilingual cognitive advantage hypothesis. We aim to specifically examine how balanced bilingualism, or the equal proficiency and usage of two languages, and the age of second language acquisition affects executive functioning in bilingual young adults.

The second goal of our study relates to the lack of research that systematically examines the effects of bilingualism on the various EF components. A widely accepted framework of EF, proposed by Miyake et al. (2000) and Friedman and Miyake (2004), consists of three core components: inhibition-related functions, mental-set shifting, and information updating and monitoring. Past studies varied in the tasks that they used to measure executive control. Some studies examined bilinguals’ advantage in the ability to inhibit interference from irrelevant information, such as the Simon task (Bialystok et al., 2004), antisaccade task (Bialystok et al., 2006b), Stroop task (Bialystok et al., 2008), or flanker task (Costa et al., 2009). Others investigated bilinguals’ advantage in tasks involving mental-set shifting, such as task-switching (Prior and MacWhinney, 2010; Barac and Bialystok, 2012). Yet other researchers focused their studies on updating information in working memory (Morales et al., 2013). Only one study has systematically and comprehensively examined the various EF components within a population of bilinguals in a single study (Soveri et al., 2011, who included four different executive control tasks; for studies that compare monolinguals and bilinguals using a comprehensive set of cognitive tasks, see Paap and Greenberg, 2013; Paap and Sawi, 2014). The results of Soveri et al.’s (2011) study suggest that bilingualism affects only the inhibition-related functions and the mental-set shifting component of executive functioning.

In sum, past studies compared different types of bilinguals against some populations of monolinguals and reported different levels of bilingual advantages across different tasks that measure different components of executive functioning. A more systematic investigation on how the degree of bilingualism would affect the various components of EF within a single bilingual population is needed in order to gain a holistic understanding of the mechanisms underlying the bilingual advantage in executive processes. Therefore, the second goal of our study is to investigate how the degree of bilingualism affects the outcomes of the various executive control components. More specifically, we are interested in whether an earlier age of L2 acquisition, a more balanced use of both languages, and/or a more balanced level of proficiency in both languages have positive effects on executive control, and whether these effects are function-general (i.e., exist across all components of executive control), or function-specific (i.e., only exist in a specific control process).

In the current study, we recruited young bilingual adults from the same population: same nationality (Singaporeans), same ethnicity (Asian), same languages acquired (English, Mandarin), same education level (undergraduates), and at the same developmentally peaked age for executive control (between 18 and 25-years-old), but varied in their age of L2 acquisition, usage and proficiency level of the two languages. Four different EF tasks were employed to represent three EF components. While it is important to acknowledge that it is difficult to measure individual components of EF due to the “*task-impurity* problem” (Burgess, 1997; Miyake and Friedman, 2012; see Jurado and Rosselli, 2007 for a complementary review), we followed Miyake et al. (2000) and Friedman and Miyake (2004) original model of EF and selected four tasks, Stroop, flanker, number–letter switching, and *n*-back, to measure the EF components of prepotent response inhibition, resistance to distractor interference, mental-set shifting, and information updating and monitoring, respectively (the first two are inhibition-related functions, see Friedman and Miyake, 2004; Miyake and Friedman, 2012).

The Stroop (1935) task is one of the most frequently used paradigms to examine prepotent response inhibition, which is the ability to suppress dominant, automatic, or prepotent responses (Miyake et al., 2000; Botvinick et al., 2001). By using a color-naming Stroop task, Bialystok et al. (2008) found bilinguals performed better than monolinguals (see Hernández et al., 2010 for a bilingual advantage in a numerical version of the Stroop task). The Eriksen flanker task (Eriksen and Eriksen, 1974) is used extensively to measure resistance to distractor interference, another inhibition-related component of EF (Friedman and Miyake, 2004). Past studies using a modified version of the flanker task (i.e., ANT) found that the bilinguals performed the task faster (Costa et al., 2009) or were less likely to be interfered by the distractors (Costa et al., 2008; Pelham and Abrams, 2014) than the monolinguals. The task-switching paradigm (Rogers and Monsell, 1995) is usually used to investigate the shifting function between different tasks or mental sets (e.g., number vs. letter; Friedman et al., 2011; Miyake and Friedman, 2012). Finally, the *n*-back task has been shown to be a valid task measuring the updating of working memory (see Kane et al., 2007).

Based on previous research that documented a strong bilingual advantage in inhibition-related functions and mental-set shifting, we predicted that an earlier second language acquisition, a more balanced use of both languages, and a more balanced level of language proficiency would result in better performance in inhibition-related tasks (e.g., a smaller interference cost in Stroop task) and mental-set shifting task (e.g., a smaller mixing cost) in the young bilingual adults.

## MATERIALS AND METHODS

### PARTICIPANTS

Seventy-two English–Mandarin young adult bilinguals (43 women, 29 men, *M*_age_ = 20.93, SD_age_ = 1.77, age range: 18–25 years) were recruited from two local public universities in Singapore. All participants were from local Singaporean families and had been living in Singapore since they were born. Singapore is a multilingual, multicultural country in Southeast Asia with English as the official language. Singapore has a bilingual policy that encourages Singaporeans to be proficient in both English and a mother tongue, which is Mandarin for the participants of this study (Silver, 2005).

Participants completed a language background questionnaire (LBQ; see Appendix A) that asked for details about each language they knew, including AoA, proficiency, and frequency of use (see Language Background Measures). All participants learned both English and Mandarin simultaneously before the age of 7 (English: *M*_AoA_ = 2.85 years, SD = 1.85, Mandarin: *M*_AoA_ = 2.44 years, SD = 1.64). Most participants acquired the languages both at home and in school (*n* = 52 for English, *n* = 62 for Mandarin). The others learned the languages either at home or in school. They also reported English and Mandarin as the two most-used languages in their present daily life; the average weekly use of English and Mandarin was 66.2% and 31.4% respectively. Of the 72 participants, 26 knew only English and Mandarin, 44 knew English, Mandarin, and a minor language(s) (either Chinese dialects such as Cantonese and Hokkien from their family members, and/or foreign languages like Japanese and German from language classes in school) but were not proficient in and did not use these minor languages regularly (average use of the minor languages was 2.5%). Another two participants reported using Cantonese regularly besides English and Mandarin (more than 20% of the time). Preliminary analyses revealed that removing these two trilingual participants did not affect the significance of the results; hence, the two participants were included in the reported analyses. **Table 1** shows the mean scores of self-rated language proficiency in English and Mandarin. The difference between self-rated English and Mandarin proficiency was significant in all aspects of language skills including comprehension, speaking, reading, and writing, indicating a bias toward English, *t*(71) > 4.07, *p*s < 0.001 (note that English is the medium of instruction for almost all subjects in Singapore schools). The average reported proficiency score for the most proficient language was 8.65 (in a 10-point scale, range = 5.75–10). There were no participants who were not proficient in both of the two languages (English and Mandarin).

**Table 1:** Mean score (and standard deviation) of self-rated proficiency^**a**^ in English and Mandarin.

	English	Mandarin	*t*	*p*
Comprehension	8.81 (1.19)	8.06 (1.45)	4.07	<0.001
Speaking	8.32 (1.40)	7.22 (1.80)	4.62	<0.001
Reading	8.56 (1.24)	7.04 (1.70)	6.52	<0.001
Writing	8.03 (1.49)	6.32 (1.81)	6.60	<0.001

*N = 72, *df* = 71 for all analyses.*

*^a^Participants rated their proficiency using a 10-point scale, where 1 indicated *not proficient* and 10 indicated *very proficient*.*

### EF TASKS: MATERIALS AND PROCEDURE

All participants completed four computerized tasks: Stroop task, Eriksen flanker task, task-switching, and *n*-back task. The tasks were programmed in MATLAB (Version 7.10) using the Psychophysics Toolbox (Version 3; Kleiner et al., 2007), and administered on a Mac mini desktop computer with a 20-inch monitor. Participants viewed the screen from a distance of about 75 cm and used a keyboard to record their responses. Instructions were presented in English at the beginning of each task and participants were instructed to respond as accurately and as quickly as possible.

#### Stroop task

A computerized version of the Stroop (1935) color-naming task was used to measure the Inhibition (Prepotent Response) component. There were three types of trials based on four colors – red, yellow, green, and blue: (a) neutral trials with a string of five asterisks printed in one of the four colors, (b) congruent trials with a color word printed in the same color (e.g., *red* printed in red), and (c) incongruent trials with a color word printed in a different color (e.g., *yellow* printed in red). The asterisks and words were displayed in 36-point Chicago font and the letters were in lower case. For all trial types, participants were instructed to respond according to the color of the font by pressing a designated key on the keyboard (*D, F, G,* and *H* for red, yellow, green, and blue, respectively). The keys were marked with matching stickers indicating the first letter of the color (*R, Y, G, B*). Each trial began with a centered white fixation cross presented against a black background for 1000 ms, followed by the stimulus that remained on the screen for a maximum of 4000 ms or until a response was made. Twelve practice trials with feedback (four trials for each trial type) were first presented to the participants, followed by 120 test trials that were divided into four blocks. All participants completed the first block consisting of 24 neutral trials. Participants then proceeded with a block of 24 congruent trials and a block of 24 incongruent trials. The test order of these two blocks was counterbalanced across participants. The last block comprised 48 mixed trials with an equal number of congruent and incongruent trials. The order of the trials within each block was randomized separately for each participant. The dependent measure was the difference in RT between the incongruent and the neutral trials, as an index of *interference* that reflects the cost of inhibiting the dominant tendency to read the word.

#### Eriksen flanker task

Adapted from Eriksen and Eriksen (1974), this is a well-accepted task that measures the Inhibition (Resistance to Distractor Interference) component. Participants were asked to decide to which direction the target (middle) arrow was pointing (left or right) while ignoring the other arrows that flanked on both sides of the target arrow. They were instructed to respond by pressing one of the arrow keys on the keyboard (← and → for left and right, respectively) using the index and ring finger of their dominant hand. In the congruent condition, all the arrows pointed to the same direction (e.g., *<<<<<*) whereas in the incongruent condition, the flankers pointed to the opposite direction of the center target arrow (e.g., *<<><<*). In addition, the difficulty of the task was manipulated by the number of flankers: (a) on easy trials, participants saw five arrows with two flankers presented on each side of the target arrow and (b) on difficult trials, participants saw nine arrows with four flankers on each side of the target arrow. The arrows subtended a visual angle of 2.62° for easy trials and 4.78° for difficult trials. On each trial, a white fixation cross first appeared in the middle of a black screen for 1000 ms, followed by a row of white arrows that remained on the screen until the participant had responded or 4000 ms had elapsed. There were eight practice trials with feedback followed by three experimental blocks with a short break in between. Participants completed a block of 40 congruent trials and then a block of 40 incongruent trials or vice versa, counterbalanced across subjects. The last block of experimental trials consisted of a mixed block with 40 congruent trials and 40 incongruent trials. The task difficulty and the direction of the target arrow were counterbalanced within each block and the order of the trials was randomized for each participant. The difference in RT between the incongruent and congruent trials was used as the dependent measure (*flanker effect*), which reflects the processing cost involved in resisting interference from distractors.

#### Task-switching (number–letter) task

In the number–letter task (adapted from Rogers and Monsell, 1995), which measures the mental-set Shifting component, participants saw a number–letter combination (e.g., *a8*) and were asked to decide whether the number was even or odd (i.e., number task) or whether the letter was a vowel or a consonant (i.e., letter task), depending on the cue that preceded the stimulus. Five consonants (*f, k, s, n, p*), five vowels (*a, e, i, o, u*), five odd digits (*1, 3, 5, 7, 9*), and five even digits (*2, 4, 6, 8, 0*) were used as the stimuli. The letters and digits were printed in 36-point Century Gothic font and the letters were in lower case. A letter was randomly paired with a digit, except the following four pairs: *o0*, *0o*, *i1,* and *1i*. In the number task, participants were instructed to press “O” on the keyboard if the digit was an odd number and “P” if it was an even number. In a letter task, participants pressed “O” if the letter was a consonant and “P” if it was a vowel. Each trial started with a centered fixation cross on screen for 1000 ms, then replaced by the task cue (the word *LETTER* or *NUMBER*) for 200 ms. Following a blank screen of 50 or 950 ms cue-stimulus interval (CSI), a number–letter pair was presented for a maximum of 5000 ms. Participants first completed two single-task blocks (i.e., a block of letter task and a block of number task, counterbalanced across participants) of 80 trials each, and then a mixed-task block of 320 trials. In the mixed-task block, half of the trials were non-switch trials, in which the current task was the same as the previous trial (e.g., *LETTER-LETTER*), and the other half were switch trials, where the current task was different from the previous trial (e.g., *LETTER-NUMBER*). The order of the trials within each block was prefixed (with the constraint that the same trial type, switch or no-switch, did not appear more than twice in a row for the mixed-task block) and was the same across all participants. There were eight practice trials to familiarize the participants with the rules of the task. Additional filler trials at the beginning of each single-task block (two trials) and the mixed-task block (four trials) were included. Within the mixed-task block, participants were given a break every 80 trials and two additional filler trials were included after each break (a total of 10 filler trials for the mixed-task block). The last filler trial was always a different task from the first experimental trial. The difference in RT between switch and non-switch trials in the mixed-task block was a measure of *switching cost*, and the difference in RT between non-switch trials in the mixed-task block and single-task trials in the single-task block was termed as *mixing cost*. The switching cost is thought to be related to more transient control processes while the mixing cost reflects global sustained control mechanisms necessary for maintaining two competing task sets (Braver et al., 2003; Rubin and Meiran, 2005).

#### N-back task

The *n*-back task (adapted from Kane et al., 2007) is a dominant measure of the Information Updating and Monitoring component. Sequences of letters were presented and participants were to indicate whether each letter was the same as the one presented two or three trials back (i.e., 2-back or 3-back task, respectively). Participants were instructed to press the spacebar when the current letter matched the *n*th-back letter. Eight phonological distinct letters (*B*, *F*, *K*, *H*, *M*, *Q*, *R*, *X*) served as target stimuli. Memory load (2-back vs. 3-back) and sequence type (no-lure vs. lure) were manipulated between blocks while stimulus type (target or foil) was manipulated within each block. In the lure condition, the eight target letters were also used as foils (e.g., the *F* in the sequence *B-F-B* is a foil in 2-back). The same target letter did not appear more than twice consecutively. In the no-lure condition, another eight letters (*U, L, G, Z, A, J, O, C*) served as foils (e.g., the sequence *B-J-G-B* in 3-back task) and the same target letter did not repeat in the subsequent trial. Stimuli were printed in 60-point Chicago font and all the letters appeared in upper case. A trial began with a centered fixation cross on screen for 500 ms, followed by the stimulus letter in the same location for another 500 ms. After an interstimulus interval of 2000 ms, a new trial was initiated. Participants first completed a 2-back and 3-back practice block of 15 trials each, and then eight experimental blocks of 24 trials each, two blocks per memory load per sequence type. In each block, there were eight target letters (25% of trials), where each target letter served as a target once, and 16 foils (75% of trials). The order of the eight blocks was randomized separately for each participant. Within each block, the order of the trials was pre-determined and remained consistent across participants. The dependent measure was the difference in the discriminability (*d*′, a measure of sensitivity in signal detection theory; see Stanislaw and Todorov, 1999) between the 3-back and 2-back tasks. The measure reflects the cost of managing the increased demands on memory updating.

### LANGUAGE BACKGROUND MEASURES

The LBQ (Appendix A) asked participants about the age they were first exposed to and the proficiency in each of the languages they know (the latter was rated on a 10-point scale where 1 is *not proficient* and 10 is *very proficient*). To obtain a measure of usage for each language, participants were first asked to estimate how often (in percent) they interact with people in different contexts (e.g., family members, colleagues, friends, or others) in a typical week and then indicate how often (in percent) they use each language in each of these contexts. The usage of the different languages in the various contexts would add up to 100%. A higher usage level in one language means a lower usage level in the other language(s). The LBQ also included questions from Rodriguez-Fornells et al. (2012) on language-switching using a 5-point scale, with higher scores indicating higher tendency to switch between languages.

### GENERAL PROCEDURE

The tasks were administered individually in a quiet room at the authors’ university. The university’s institutional review board approved the study. All participants provided informed consent before participating in the study. Participants completed the four EF tasks, followed by the LBQ. The order of the four EF tasks was counterbalanced across participants based on a Latin square design.

## RESULTS

### LANGUAGE BACKGROUND MEASURES ANALYSES

Demographic information and mean scores on the language background measures are presented in **Table 2**. For most participants (*n* = 65), their most proficient language was also the one they used most often. However, for a small number of the participants (*n* = 7), the most proficient language was not the one that they used most often. Similarly, for some participants (*n* = 9), the first language they acquired was not the same language that they used most often or were most proficient in. As such, for each participant, we first calculated the AoA, usage, as well as proficiency scores of each of the two languages. The following variables were then determined: AoA_1_ (age of L1 acquisition, i.e., age at which the first language was acquired), AoA_2_ (age of L2 acquisition, i.e., age at which the second language was acquired), Usage_1_ (the higher usage score of the two languages), Usage_2_ (the lower usage score of the two languages), Proficiency_1_ (the higher proficiency score of the two languages), and Proficiency_2_ (the lower proficiency score of the two languages).

**Table 2:** Demographics, mean score, and standard deviation on language background measures.

	Mean	SD
Age of L1 acquisition (in years)	2.15	1.51
Age of L2 acquisition (in years)	3.14	1.85
Usage_1_^a^	0.74	0.15
Usage_2_^a^	0.24	0.14
Proficiency_1_^b^	8.65	1.11
Proficiency_2_^b^	6.94	1.43
Balanced usage (Usage_1_ – Usage_2_)	0.50	0.28
Balanced proficiency (Proficiency_1_ – Proficiency_2_)	1.70	1.37
Language-switching^c^	24.21	5.79

*N = 72.*

*^a^Self-reported proportion of language use weekly in various contexts (e.g., if a participant reported using a language 80% of the time weekly, the usage score would be 0.80 for this language). Usage_1_ is the higher usage score and Usage_2_ is the lower usage score of the two languages.*

*^b^Average of self-reported proficiency in language comprehension, speaking, reading and writing, ranging from 1 = *not proficient* to 10 = *very proficient*. Proficiency_1_ is the higher proficiency score and Proficiency_2_ is the lower proficiency score of the two languages.*

*^c^Total score of 9 questions on the frequency of language-switching, ranging from 1 = never to 5 = always.*

In order to investigate how acquiring a second language early and how balanced bilingualism affect executive functioning, we decided to use the following three variables as indicators of bilingualism in our analyses: (1) AoA_2_ – the age of L2 acquisition, indicating the onset of bilingualism, (2) balanced usage (Usage_1_ – Usage_2_), as an indicator of balanced bilingualism relating to language use, and (3) balanced proficiency (Proficiency_1_ – Proficiency_2_), as an indicator of balanced bilingualism relating to language competency. For the balanced usage and proficiency scores, a minimum score of 0 indicates perfect balance in the two languages. Therefore, the higher the score, the less balanced a bilingual participant is reported to be. Do note, however, that the participants in our study were proficient in at least one language (score of 5 and above, out of 10). There was no participant who was not proficient in both of the two languages.

**Table 3** shows the correlations between the three bilingualism indicators and self-reported language-switching behavior. There was a significant relationship between the two indices of balanced bilingualism, balanced usage, and balanced proficiency, *r* = 0.56, *p* < 0.001, *d* = 1.35, indicating that participants who use two languages regularly also tend to be equally proficient in the two languages. Language-switching was also significantly correlated with balanced usage and balanced proficiency, *r* = –0.44, *p* < 0.001, *d* = 0.98, and *r* = –0.32, *p* = 0.007, *d* = 0.68, respectively. This suggests that participants switch languages more frequently if they are more balanced in their two languages, which supported the notion that bilinguals switch between languages because they have the competency to do so in both languages (Muysken, 2000; Poplack, 2001; Yow et al., in press). However, AoA_2_ was not correlated with balanced usage (*r* = –0.19, *p* = 0.12, *d* = 0.39) or balanced proficiency (*r* = 0.06, *p* = 0.60, *d* = 0.12). The onset of bilingualism may not be related to the regular usage of and the equivalent proficiency in the two languages. Early bilingualism in childhood does not necessarily indicate balanced bilingualism in adulthood.

**Table 3:** Correlation matrix for the language background measures.

	1	2	3	4
(1) AoA of L2	–			
(2) Balanced usage	–0.19	–		
(3) Balanced proficiency	0.06	0.56***	–	
(4) Language-switching	–0.04	–0.44***	–0.32**	–

N = 72.

**p < 0.01, ***p < 0.001.

### EF TASK ANALYSES

Two participants were each missing data for two EF tasks (Stroop and flanker) because of equipment malfunction. Another seven participants were excluded from the analyses for one EF task due to low accuracy, that is, their individual task accuracy means were 2.5 SD below the overall mean of the task (*n* = 1 for flanker, *n* = 4 for *n*-back, *n* = 2 for task-switching). There were 70 participants in the final sample for both Stroop and task-switching. For the flanker and *n*-back task, 69 and 68 participants were included in the reported analyses, respectively. For the final sample, average accuracy was greater than 89% for all the EF tasks.

For the RT measures (except *n*-back, which did not depend on a mean RT), incorrect responses, trials with RTs less than 200 ms and trials with RTs more than 2.5 SD from the mean in each condition (e.g., congruent or incongruent trials in the flanker task) were discarded for each participant. This allows for the best measure of central tendency for each condition to be obtained (Friedman et al., 2011). The percentage of the eliminated trials was less than 7.8% for all of the tasks. RT measures were assessed using repeated-measures analyses of variance (ANOVAs) to examine the cost of processing for each EF task. The key variables of the respective EF tasks were significant, indicating robust processing costs as expected of each task (see Appendix B).

### EFFECTS OF BILINGUALISM ON EF: REGRESSION ANALYSES

To examine how bilingualism affects executive functioning, multiple linear regression analyses were conducted separately for each of the processing costs associated with the respective EF task. The three indicators of bilingualism (AoA of L2, balanced usage, and balanced proficiency) were predictors of the processing cost. As balanced usage and balanced proficiency were significantly correlated with each other, which may lead to multicollinearity (Dormann et al., 2013), separate models were created for their effect on each EF task, in order to produce more feasible and interpretable models (Kuhn and Johnson, 2013). Thus, for each EF task, two of the three predictors were entered simultaneously into the model: (1) AoA of L2 and balanced usage, and (2) AoA of L2 and balanced proficiency (see Appendix C). We predicted that an earlier acquisition of L2, a more balanced usage of and a more balanced proficiency in the two languages would result in a better performance in inhibition-related and mental-set shifting tasks (provided participants are proficient in at least one of the two languages).

#### Interference effect in Stroop

The model with AoA of L2 and balanced usage was significant. The two predictors explained 12% of the variance, *F*(2,67) = 4.73, *p* = 0.012, *R*^2^ = 0.12, *R*^2^_adjusted_ = 0.10. Most importantly, it was found that the interference effect was significantly predicted by AoA of L2, β = 0.27, *t*(67) = 2.35, *p* = 0.022, as well as balanced usage, β = 0.28, *t*(67) = 2.40, *p* = 0.019. This demonstrates that an earlier age of L2 acquisition and a more balanced use of two languages resulted in a smaller interference effect in the Stroop task. The regression model with AoA of L2 and balanced proficiency was nearly significant for the Stroop interference effect, *F*(2,67) = 2.89, *p* = 0.063, *R*^2^ = 0.08, *R*^2^_adjusted_ = 0.05. The effect of AoA of L2 was found to be marginally significant, β = 0.21, *t*(67) = 1.76, *p* = 0.084, but balanced proficiency did not significantly predict the interference effect, β = 0.18, *t*(67) = 1.50, *p* = 0.14. In sum, AoA of L2 and balanced usage had a significant impact on the interference effect in the Stroop task.

#### Mixing cost in task-switching

The regression model with the two predictors, AoA of L2 and balanced usage was significant for the mixing cost in task-switching and explained 15% of the variance, *F*(2,67) = 5.85, *p* = 0.005, *R*^2^ = 0.15, *R*^2^_adjusted_ = 0.12. The mixing cost was significantly predicted by balanced usage, β = 0.39, *t*(67) = 3.41, *p* = 0.001, but not AoA of L2. Similar to the results in the Stroop task, this suggests that the more a bilingual participant uses both languages, the smaller the mixing cost he or she experiences in the number–letter task. The model for the mixing cost was also significant with the two predictors, AoA of L2 and balanced proficiency, and the two predictors explained 13% of the variance, *F*(2,67) = 4.86, *p* = 0.011, *R*^2^ = 0.13, *R*^2^_adjusted_ = 0.10. The mixing cost was significantly predicted by balanced proficiency, β = 0.36, *t*(67) = 3.11, *p* = 0.003, but not AoA of L2, indicating that bilinguals who were more equally proficient in their two languages demonstrated smaller mixing costs. In sum, both balanced usage and balanced proficiency were significant predictors of the mixing cost in task-switching, even when controlling for AoA of L2.

The regression models for the flanker effect, switching cost and the *n*-back effect were not significant (L2 AoA and balanced usage: *F*(2,66) = 0.92, *R*^2^ = 0.03 for flanker effect; *F*(2,67) = 0.37, *R*^2^ = 0.01 for switching cost; *F*(2,65) = 0.17, *R*^2^ = 0.01 for *n*-back effect, all *ps* > 0.10; L2 AoA and balanced proficiency: *F*(2,66) = 1.71, *R*^2^ = 0.05 for flanker effect; *F*(2,67) = 0.86, *R*^2^ = 0.03 for switching cost; *F*(2,65) = 0.13, *R*^2^ = 0.004 for *n*-back effect, all *ps* > 0.10). In summary, the regression models were significant only for the Stroop interference effect and the mixing cost. The statistics of the regression model and standard coefficients for each predictor are shown in Appendix C.

### EFFECTS OF BILINGUALISM ON EF: STRUCTURAL EQUATION MODELING AND BOOTSTRAPPING

The results of the regression analyses showed that both the Stroop interference effect and the mixing cost in task-switching were affected by at least one of the three indicators of bilingualism (onset of bilingualism, balance in language use, and balance in language proficiency). However, since balanced usage and balanced proficiency were significantly correlated with each other, it is possible that both variables contribute to the same underlying construct *balanced bilingualism*. We decided to test this hypothesis using structural equation modeling (SEM), which allows us to simultaneously consider the effects of the two correlated variables via a latent variable, balanced bilingualism.

**FIGURE 1:**
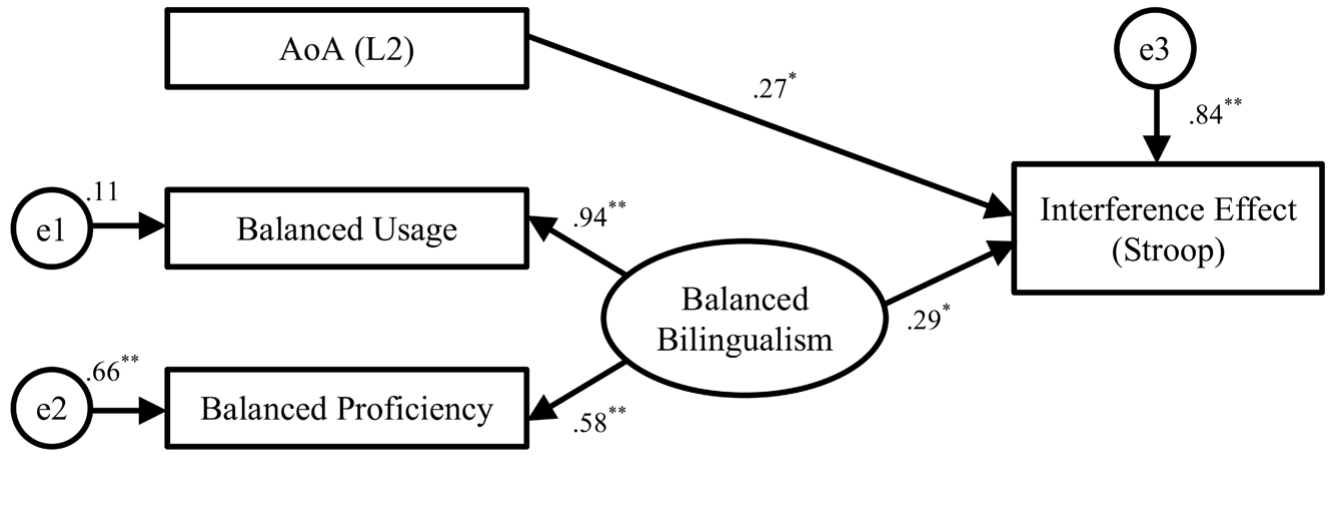
**Structural equation model of the interference effect in Stroop task.** *N* = 70. The standardized maximum likelihood parameter estimates are shown. The error variances (e1, e2, and e3) indicate the amount of unexplained variance. Thus, for the observed variable of Balance Usage, Balanced Proficiency and Interference Effect, *R*^*2*^ = (1 - error variance). *p < 0.05, **p < 0.01.

We first estimated the model for the interference effect in Stroop. The hypothesized model, as shown in **Figure 1**, depicted the directional relationships between the exogenous variables (AoA of L2, balanced usage, and balanced proficiency), the latent variable (balanced bilingualism), and the endogenous outcome variable (interference effect). The model was fitted using the method of maximum likelihood in SPSS Amos 21 (see Appendix D for the descriptive statistics and correlation matrices of the SEMs). According to McDonald and Ho (2002) and Kline (2005), some of the most used fit indices and their acceptable thresholds include: (1) χ^2^ (the lower the χ^2^, the better the model’s fit), (2) goodness of fit index (GFI; values greater than 0.90 indicate a good fit), (3) comparative fit index (CFI; values close to 0.93 indicate a good fit), and (4) the standardized root mean square residual (sRMR; a value close to or below 0.08 is generally considered favorable).

Based on these criteria, the hypothesized model for the Stroop interference effect produced a reasonable fit to the data, χ^2^(2) = 5.99, *p* = 0.06, GFI = 0.96, CFI = 0.90; sRMR = 0.067. In addition, 16% of the variance in the interference effect was explained by the three exogenous variables. The major focus of the model is the path regression coefficients from the bilingualism indicators (L2 AoA and balanced bilingualism) to the endogenous outcome (interference effect). As seen in **Figure 1**, the path from L2 AoA to the interference effect was significant; suggesting that early onset of bilingualism is associated with smaller processing cost in the Stroop task. More importantly, the latent variable of balanced bilingualism also showed a significant relationship with the interference effect, such that the more balanced bilinguals performed better in the Stroop task with a smaller interference effect than the less balanced bilinguals.

**FIGURE 2:**
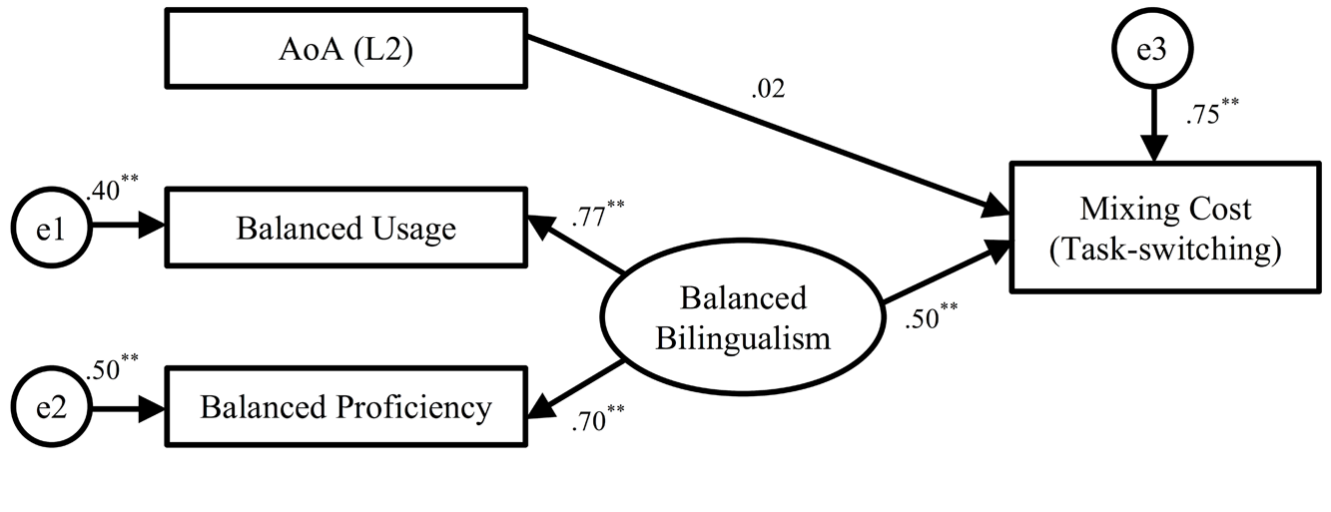
**Structural equation model of the mixing cost in task-switching.** *N* = 70. The standardized maximum likelihood parameter estimates are shown. The error variances (e1, e2, and e3) indicate the amount of unexplained variance. Thus, for the observed variable of Balance Usage, Balanced Proficiency and Mixing Cost, *R*^*2*^ = (1 - error variance). **p < 0.01.

We next tested the model for the mixing cost in task-switching using the same method. **Figure 2** illustrates the model with mixing cost as the endogenous outcome variable. The hypothesized model produced a reasonable fit to the data, χ^2^(2) = 5.77, *p* = 0.06; GFI = 0.96; CFI = 0.90; sRMR = 0.065. The exogenous variables of L2 AoA and balanced bilingualism explained 25% of the variance in the endogenous variable of mixing cost. The regression coefficient of the path from the latent variable balanced bilingualism to the outcome variable of mixing cost was significant (but not L2 AoA). The more balanced the bilinguals are in terms of usage and proficiency in the two languages, the smaller the mixing cost they tend to incur.

Lastly, we retested the parameter estimates in the above SEMs using bootstrapping procedures. The Bollen–Stine bootstrap was applied to get a bootstrap adjusted *p* value, which tested the null hypothesis that the model was correct (Bollen and Stine, 1992; Nevitt and Hancock, 2001). Thus, a Bollen–Stine bootstrap *p* value larger than 0.05 would indicate a good fit of the model. For each model, the Amos program generated 1,000 bootstrap samples automatically. The results of the bias-corrected significance tests were consistent with the SEM results. The Bollen–Stine *p* values were 0.060 and 0.085 for the models of interference effect and mixing cost, respectively, indicating that the two models fit well to the data (a summary of the bootstrap estimates for the standardized regression weights and confidence intervals is shown in Appendix E).

The data from bootstrapping SEMs further supported the results of the regression analyses and we were able to consider the two correlated variables, balanced usage and balanced proficiency, simultaneously by measuring their common factor that was defined as balanced bilingualism. In sum, our results suggested that balanced bilingualism had a significant impact on both response inhibition as well as mental-set shifting. The onset of bilingualism also played an important role in bilinguals’ inhibition of prepotent responses.

## DISCUSSION

The current study seeks to test the underlying mechanisms of the bilingual cognitive advantage hypothesis amidst recent findings of inconsistent results in between-group performances of monolinguals and bilinguals. Specifically, we considered bilingualism as a continuous variable and examined whether an earlier age of second language acquisition, and a more balanced use and a more balanced level of proficiency of two languages would have significant effects on some or all EF components. This rests on the basis that the bilinguals are proficient in at least one of the two languages. In a sample of 72 English–Mandarin early bilinguals, we found that the extent of balanced bilingualism (usage and proficiency) and the age of L2 acquisition could predict individual performance in some tasks involving executive control. In particular, age of L2 acquisition was associated positively with the interference cost in the prepotent response inhibition task (Stroop). The earlier the bilinguals acquired the second language, the better they were at inhibiting prepotent responses in the task. More importantly, there was a significant effect of the latent variable balanced bilingualism (balanced usage and proficiency in two languages) on certain components of executive functioning. Bilinguals who used both languages frequently and have comparable levels of proficiency in both languages tended to have a smaller interference effect in the prepotent response inhibition task and a smaller mixing cost in the mental-set shifting task (task-switching). These effects were replicated with a sample of 1,000 using bootstrapping procedures.

Past research that compares the cognitive performance of monolinguals and bilinguals has regarded monolingualism and bilingualism as discrete all-or-none variables. Some studies recruited bilinguals who used both languages regularly (Bialystok et al., 2005a, 2006a; Bialystok and Feng, 2009), while others included bilinguals who were either equally proficient in the two languages (Bialystok et al., 2006a; Salvatierra and Rosselli, 2010; Iluz-Cohen and Armon-Lotem, 2013), or had an early onset of bilingualism (Bialystok and Feng, 2009; Luk et al., 2011; Kapa and Colombo, 2013). They found that these bilinguals performed better in some executive control tasks compared to a group of monolinguals. Yet using seemingly similar criteria, several recent studies did not find a bilingual advantage across multiple executive control tasks, even after matching samples of monolinguals and bilinguals with respect to important demographics characteristics (e.g., Paap and Greenberg, 2013; Antón et al., 2014; Gathercole et al., 2014; Paap and Sawi, 2014). However, the process of determining who is a monolingual and who is a bilingual remains constrained by the rules each study applied. For example, Salvatierra and Rosselli (2010) selected participants as bilinguals if they rated themselves 3 out of 5 as being able to understand and speak both languages well and participants as monolinguals if they did not know a second language at all. In comparison, Paap and Greenberg classified participants as bilingual if they self-rated at least a 4 (out of a 7-point scale) in proficiency for both languages. Participants in their study were considered monolingual if they self-rated a 3 or less for one language, even if they self-rated a 4 or above in proficiency for the other language. It is possible that the mixed findings of a bilingual cognitive advantage are due to the nuances lost when fitting individuals into groups of either monolinguals or bilinguals. We believe that considering monolingualism-bilingualism as a variable that falls along a continuum rather than as a variable that categorizes participants based on some arbitrary rules would help in resolving these inconsistencies.

Our findings also propose that bilinguals’ regular and extensive experience in controlling attention to their two language systems results in better executive functioning in some components such as inhibiting prepotent responses and shifting attention, but not in other components such as resistance to distractor or information updating and monitoring. This task difference suggests that the impact of bilingualism on executive control is function-specific and not function-general; that is, the bilingual effect may not exist across all components of EF. The regular practice of controlling and attending to two language systems appears to be more related to the executive processes involved in inhibiting prepotent responses (e.g., interference effect in a Stroop task) and controlling global set-shifting (e.g., mixing cost in the number–letter switching task) than resisting interference from distractors (e.g., flanker effect) and updating information from memory (e.g., *n*-back effect). One possible explanation for this function specificity is that balanced bilinguals have to engage in the control of *two* sets of (language) rules more often (similar demands required in the Stroop and number–letter switching task) but not required to ignore distractors or manage increased demands from the *same* (language) system more often than the less balanced bilinguals. In other words, since flanker and *n*-back task do not require one to manage two different sets of rules at the same time, bilingualism may have less of an advantage in this component.

This finding may be limited to the specific experimental design and tasks selected to test the different EF components, however. For example, we found the effects of balanced bilingualism in mixing cost but not in switching cost. This finding is consistent with Soveri et al. (2011) but is different from Prior and colleagues (Prior and MacWhinney, 2010; Prior and Gollan, 2011; see Hernández et al., 2013 for a summary of studies comparing bilinguals and monolinguals in the task-switching paradigms). Both Soveri et al. (2011) and our study used a number–letter task while Prior and colleagues (Prior and MacWhinney, 2010; Prior and Gollan, 2011) used a color-shape task. Perhaps tasks involving verbal stimuli are recruiting language control processes rather than executive control processes (Hernández et al., 2010). Bilinguals who acquired English earlier or have a higher level of English proficiency might be better at performing tasks that involved verbal stimuli than those who acquired English later or with a lower level of English proficiency. *Post hoc* analyses revealed that neither English AoA nor English proficiency was correlated with the task performance in the three tasks where verbal materials were used (Stroop, task-switching, and *n*-back task; see Appendix F). Thus, the difference in findings may not be due to the difference in stimuli used. In addition, bilinguals were compared with monolinguals in Prior’s studies but we examined performance within a group of bilinguals with variable experiences in bilingualism. How participants are selected and with whom they are compared may be the key reason to why our results differ from Prior and Gollan’s (2011). Future studies could examine whether inconsistent findings are due to task stimuli or participants or both.

It has been argued that EFs are not neatly separated into distinct components. A recent theoretical view about EFs (Miyake and Friedman, 2012), which proposed a unity and diversity framework of EF components, makes it more challenging to investigate the components individually. Differences in task-specific performance on the various measures of executive functioning could also indicate a lack of convergent validity in inhibitory control, switching, and monitoring (Paap and Greenberg, 2013; Paap and Sawi, 2014). It is nonetheless important to determine how bilingualism affects performance in the various EFs so that we can better understand the unique contribution of language experience to cognitive outcomes. Future studies may be replicated using other executive tasks to examine the robustness of the effects we have found in this study.

Executive functions are critical for many of the skills that are important for success in life, such as mental and physical health, school readiness and success, career achievement, marital harmony, and public safety (see Diamond, 2013, p. 137, for a review). Recent research suggests that EFs can be taught and improved with training (e.g., Klingberg, 2010; Bergman Nutley et al., 2011; Diamond and Lee, 2011). However, the magnitude and generalizability of the improvements greatly depends on the type of training tasks and the amount of time spent working on these skills. Those studies with the most encouraging gain usually involve intensive daily engagement in activities that train and challenge executive functioning in multiple ways (Klingberg et al., 2005; Lillard and Else-Quest, 2006; Diamond et al., 2007; Karbach and Kray, 2009). Being a balanced bilingual is akin to be trained regularly in executive control skills, such as inhibiting prepotent responses and task switching. In fact, previous work that found a bilingual advantage in executive control skills indicates that bilingualism may be a unique type of *executive function training* that is successful in transferring language management skills to the global measures of executive functioning. Given that a balanced dual-language environment is both immersive and extensive, balanced bilingualism may play an even greater role in the promotion and development of executive control skills.

This study fills an important gap in our understanding of the effects of bilingualism on the various EFs and challenges how future studies should construe bilingualism as a variable. Our study provides evidence that bilinguals do derive cognitive benefits in some executive control processes from their regular practice of the two language systems. We have shown that the more balanced a bilingual is (in terms of the usage of and the proficiency in two languages), the better his or her inhibitory control and global set-shifting skills would be, provided that he or she is at least moderately proficient in one language. Early bilinguals (those who have acquired two languages early) also benefitted from such early exposure, which would have provided more time for them to engage in the regular practice of the two language systems. Thus, it is the constant practice of controlling and attending to two language systems that is pertinent to the development of specific executive control skills in bilingual young adults, which may have contributed to the bilingual advantage in these skills compared to their monolinguals peers over time.

## SUPPLEMENTARY MATERIAL

The Supplementary Material for this article can be found online at: http://www.frontiersin.org/journal/10.3389/fpsyg.2015.00164/abstract

## Conflict of Interest Statement

The authors declare that the research was conducted in the absence of any commercial or financial relationships that could be construed as a potential conflict of interest.
