# Supplementary material for: Balanced bilingualism and early age of second language acquisition as the underlying mechanisms of a bilingual executive control advantage: why variations in bilingual experiences matter
Source: Front Psychol. 2015 Feb 26;6:164. doi: 10.3389/fpsyg.2015.00164 (PMC4341428; doi:10.3389/fpsyg.2015.00164)
Supplement: Supplementary file 1 [file table_1.docx]

Appendix A

Language Background Questionnaire

## 1. LANGUAGE BACKGROUND

1. Write down all the **language(s)** that you know, the approximate **age** that you **first** learn each of them.

| Language | Age first acquired |
| --- | --- |
| 1. |  |
| 2. |  |
| 3. |  |
| 4. |  |

1. For each of the above language(s), **using the scale below**, rate how **proficient** you are in each of them:

| Language Proficiency |
| --- |
| 1 2 3 4 5 6 7 8 9 10  Not Moderately Very  proficient proficient proficient |

|  | Language Proficiency | | | |
| --- | --- | --- | --- | --- |
| Language | Understand (Listening) | Understand (Reading) | Speak | Write |
| 1. |  |  |  |  |
| 2. |  |  |  |  |
| 3. |  |  |  |  |
| 4. |  |  |  |  |

1. For each of the above language(s), from whom or where did you learn the language? (You can check more than one)

| Language | How did you learn the language? |
| --- | --- |
| 1. | Mother Father Care- School Lang. Others:  giver Classes Click here to enter text. |
| 2. | Mother Father Care- School Lang. Others:  giver Classes Click here to enter text. |
| 3. | Mother Father Care- School Lang. Others:  giver Classes Click here to enter text. |
| 4. | Mother Father Care- School Lang. Others:  giver Classes Click here to enter text. |

2. PAST LANGUAGE USAGE

1. For each of the above language(s), **using the scale below**, approximately rate how **often** you use it during **each** time period in your **past**:

| Frequency of Language Use |
| --- |
| 0 1 2 3 4 5 6 7 8 9 10 NA  Nil Occasionally Moderately Very Often  (less than once a month) (a couple of times a month) (everyday) |

| Language | 0 – 3  years old | 4 – 7  years old | 8 – 12 years old | 13 – 18 years old | 19 – 30 years old |
| --- | --- | --- | --- | --- | --- |
| 1. |  |  |  |  |  |
| 2. |  |  |  |  |  |
| 3. |  |  |  |  |  |
| 4. |  |  |  |  |  |

3. CURRENT LANGUAGE USAGE

| (a) For a **typical week**, estimate how much **time (%)** you spend with the following people (the interactions can include face-to-face, phone/skype call, emailing, instant message, sms, social networking at any time and at any places, such as at school, dorms, home, or off campus): (they should add up to 100%) | (b) For **each context**, estimate how much **time (%)** you spend **using** each of the above **language(s):** (they should add up to 100%) |
| --- | --- |
| Context | Language |
| With family members % | \| **Language** \| **Percentage (%)** \| \| --- \| --- \| \| 1. \|  \| \| 2. \|  \| \| 3. \|  \| \| 4. \|  \| |
| With friends % | \| **Language** \| **Percentage (%)** \| \| --- \| --- \| \| 1. \|  \| \| 2. \|  \| \| 3. \|  \| \| 4. \|  \| |
| Others %  please specify the context: | \| **Language** \| **Percentage (%)** \| \| --- \| --- \| \| 1. \|  \| \| 2. \|  \| \| 3. \|  \| \| 4. \|  \| |
| Total = 100 % |  |

4. LANGUAGE-SWITCHING

Please, try to answer to what degree the following questions are representative of the manner you use to talk or speak in the languages you know (e.g., Mandarin, English, Malays, Hokkien etc). Many of these questions ask you to report your tendency to switch or mix languages during a conversation. Switching and mixing languages is a characteristic of some bilingual contexts or environments, as for example in Singapore. The present questionnaire aims to identify the language switching patterns that exist in these languages. If you have doubts about how to rate yourself in the following questions, please try to compare your manner of speaking and talking with that of most people, or those who you know very well.

| 1 | 2 | 3 | 4 | 5 |
| --- | --- | --- | --- | --- |
| Never | Very infrequently | Occasionally | Frequently | Always |

1. I do not remember or I cannot recall some words in a particular language when I am speaking in this language.Choose an item.
2. I tend to switch languages during a conversation (for example, I switch from English to Mandarin or vice versa). Choose an item.
3. When I cannot recall a word in one language, I tend to immediately produce it in another language. Choose an item.
4. I do not realize when I switch the language during a conversation (e.g., from English to Mandarin or etc.) or when I mix languages; I often realize it only if I am informed of the switch by another person. Choose an item.
5. When I switch languages, I do it consciously. Choose an item.
6. It is difficult for me to control the language switches I introduce during a conversation (e.g., from English to Mandarin, vice-versa or etc.) Choose an item.
7. Without intending to, I sometimes produce the word in other language(s) faster when I am speaking in one language. Choose an item.
8. There are situations in which I always switch between the two or more languages. Choose an item.
9. There are certain topics or issues for which I normally switch between languages. Choose an item.

Appendix B

Table B1 Descriptive Statistics of the Processing Cost in Each of the Executive Function Tasks

|  |  | Control Trial | |  | EF Trial | |  | Difference | *F* | *p* | Effect Size | |
| --- | --- | --- | --- | --- | --- | --- | --- | --- | --- | --- | --- | --- |
|  |  | *M* | *SD* |  | *M* | *SD* |  |  |  |  |  |  |
|  |  | Neutral | |  | Incongruent | |  | Interference  effect | | | | |
| Stroop | RT (ms) | 684 | 123 |  | 792 | 171 |  | 107 | 59.13 | < .001 | | .461 |
|  |  | Congruent | |  | Incongruent | |  | Flanker  effect | | | | |
| Flanker | RT (ms) | 443 | 89 |  | 606 | 168 |  | 164 | 141.28 | < .001 | .675 | |
|  |  | Non-switch (mixed-task) | |  | Switch  (mixed-task) | |  | Switching  cost | | | | |
| Task-switching | RT (ms) | 982 | 222 |  | 1040 | 278 |  | 58 | 22.81 | < .001 | .248 | |
|  |  | Non-switch (single-task) | |  | Non-switch (mixed-task) | |  | Mixing  cost | | | | |
|  | RT (ms) | 783 | 147 |  | 982 | 222 |  | 199 | 130.68 | < .001 | .654 | |
|  |  | 2-back | |  | 3-back | |  | *N*-back  effect | | | | |
| *N*-back | *d*' | 3.17 | 0.53 |  | 2.30 | 0.52 |  | 0.87 | 228.22 | < .001 | .773 | |

*Note.* *n* = 70 for Stroop task, *n* = 69 for flanker task, *n* = 70 for task-switching, and *n* = 68 for *n*-back task.

Appendix C

Table C1 Summary of Multiple Regression Analyses

| Variables | Stroop interference  (β) | Flanker effect  (β) | Switching cost  (β) | Mixing  cost  (β) | *N*-back effect  (β) |
| --- | --- | --- | --- | --- | --- |
| Model 1: Performance predicted by AoA of L2 and Balanced Usage | | | | | |
| AoA of L2 | .27^*^ | − .06 | − .10 | .05 | .02 |
| Balanced Usage | .28^*^ | − .16 | .02 | .39^**^ | − .07 |
| *R*^2^ | .12 | .03 | .01 | .15 | .01 |
| *F* | 4.73^*^ | 0.92 | 0.37 | 5.85^**^ | 0.17 |
| Model 2: Performance predicted by AoA of L2 and Balanced Proficiency | | | | | |
| AoA of L2 | .21^+^ | .02 | − .11 | − .05 | .03 |
| Balanced Proficiency | .18 | − .22 | .12 | .36^**^ | − .06 |
| *R*^2^ | .08 | .05 | .03 | .13 | .004 |
| *F* | 2.89^+^ | 1.71 | 0.86 | 4.86^*^ | 0.13 |

*Note*. *n* = 69 for flanker effect, *n* = 68 for *n*-back effect, *n* = 70 for all the other costs. ^+^*p* < .10 ^*^*p* < .05, ^**^*p* < .01.

Appendix D

Table D1 Descriptive Statistics and Correlations Matrices for the Observed Variables in SEMs

|  |  | 1 | 2 | 3 | 4 | *M* | *SD* |
| --- | --- | --- | --- | --- | --- | --- | --- |
| SEM for Interference Effect in Stroop | 1. AoA of L2 | − |  |  |  | 3.10 | 1.86 |
|  | 2. Balanced Usage | -.19 | − |  |  | 0.50 | 0.28 |
|  | 3. Balanced Proficiency | .08 | .55^***^ | − |  | 1.72 | 1.38 |
|  | 4. Interference Effect | .22^†^ | .22^*^ | .19 | − | 107.44 | 116.90 |
| SEM for Mixing Cost in task-switching | 1. AoA of L2 | − |  |  |  | 3.10 | 1.86 |
|  | 2. Balanced Usage | −.21 | − |  |  | 0.50 | 0.28 |
|  | 3. Balanced Proficiency | .05 | .54^***^ | − |  | 1.72 | 1.38 |
|  | 4. Mixing Cost | -.03 | .38^**^ | .35^**^ | − | 199.25 | 145.82 |

*Note*. *N* = 70 for each model.

^†^*p* < .10, ^*^*p* < .05, ^**^*p* < .01, ^***^*p* < .001.

Appendix E

Table E1 Estimates of Individual Paths in SEMs Using Bootstrap Method

| Model | Parameter |  | *Bootstrap* (1,000 bootstrap samples) | | | | |
| --- | --- | --- | --- | --- | --- | --- | --- |
|  |  |  | β | *SE* | 95% bias-corrected confidence interval | | *p* |
|  |  |  |  |  | Lower | Upper |  |
| Interference Effect in Stroop | Balanced Usage 🡨 BB |  | .937 | .007 | .567 | 2.084 | <.001 |
|  | Balanced Proficiency 🡨 BB |  | .630 | .004 | .254 | .869 | .009 |
|  | Interference Effect 🡨 AoA (L2) |  | .256 | .002 | .020 | .460 | .031 |
|  | Interference Effect 🡨 BB |  | .325 | .003 | .072 | .544 | .030 |
| Mixing Cost in task-switching | Balanced Usage 🡨 BB |  | .815 | .005 | .513 | 1.209 | .004 |
|  | Balanced Proficiency 🡨 BB |  | .693 | .003 | .421 | .964 | .002 |
|  | Mixing Cost 🡨 AoA (L2) |  | .015 | .003 | −.225 | .301 | .856 |
|  | Mixing Cost 🡨 BB |  | .481 | .003 | .182 | .722 | .002 |

*Note*. *N* = 70 for each model. BB = the latent variable of Balanced Bilingualism.

Appendix F

Table F1 Correlations between English AoA or English Proficiency and Task Performance in Stroop, Task-Switching, and *N*-back

|  | Stroop interference | Switching cost | Mixing cost | *N*-back effect |
| --- | --- | --- | --- | --- |
| English AoA | .17 | - .08 | - .07 | .01 |
| English Proficiency | - .18 | - .05 | - .07 | - .11 |

*Note*. *n* = 70 for Stroop and task-switching, *n* = 68 for *n*-back.
